# Similar Prevalence of Low-Abundance Drug-Resistant Variants in Treatment-Naive Patients with Genotype 1a and 1b Hepatitis C Virus Infections as Determined by Ultradeep Pyrosequencing

**DOI:** 10.1371/journal.pone.0105569

**Published:** 2014-08-20

**Authors:** Severine Margeridon-Thermet, Sophie Le Pogam, Lewyn Li, Tommy F. Liu, Nancy Shulman, Robert W. Shafer, Isabel Najera

**Affiliations:** 1 Infectious Diseases and Geographic Medicine, Stanford University Medical School, Stanford, California, United States of America; 2 Infectious Diseases Discovery, Pharma Research Early Development, Roche, Nutley, New Jersey, United States of America; 3 Pharma Development, Genentech, South San Francisco, California, United States of America; 4 Infectious Diseases Discovery, Pharma Research Early Development, Roche, Shanghai, China; Rutgers, The State University of New Jersey, United States of America

## Abstract

**Background and Objectives:**

Hepatitis C virus (HCV) variants that confer resistance to direct-acting-antiviral agents (DAA) have been detected by standard sequencing technology in genotype (G) 1 viruses from DAA-naive patients. It has recently been shown that virological response rates are higher and breakthrough rates are lower in G1b infected patients than in G1a infected patients treated with certain classes of HCV DAAs. It is not known whether this corresponds to a difference in the composition of G1a and G1b HCV quasispecies in regards to the proportion of naturally occurring DAA-resistant variants before treatment.

**Methods:**

We used ultradeep pyrosequencing to determine the prevalence of low-abundance (<25% of the sequence reads) DAA-resistant variants in 191 NS3 and 116 NS5B isolates from 208 DAA-naive G1-infected patients.

**Results:**

A total of 3.5 million high-quality reads of ≥200 nucleotides were generated. The median coverage depth was 4150x and 4470x per NS3 and NS5B amplicon, respectively. Both G1a and G1b populations showed Shannon entropy distributions, with no difference between G1a and G1b in NS3 or NS5B region at the nucleotide level. A higher number of substitutions that confer resistance to protease inhibitors were observed in G1a isolates (mainly at amino acid 80 of the NS3 region). The prevalence of amino acid substitutions that confer resistance to NS5B non-nucleoside inhibitors was similar in G1a and G1b isolates. The NS5B S282T variant, which confers resistance to the polymerase inhibitors mericitabine and sofosbuvir, was not detected in any sample.

**Conclusion:**

The quasispecies genetic diversity and prevalence of DAA-resistant variants was similar in G1a and G1b isolates and in both NS3 and NS5B regions, suggesting that this is not a determinant for the higher level of DAA resistance observed across G1a HCV infected patients upon treatment.

## Introduction

Advances in the knowledge of the structure and function of hepatitis C virus (HCV) proteins and the development of robust methods for studying HCV replication *in* *vitro* have resulted in the development of direct acting antiviral agents (DAAs) that target essential proteins, primarily the NS3/4A serine protease, the RNA dependent RNA polymerase (RdRp, NS5B) and NS5A. Three agents that inhibit the NS3/4A serine protease are now approved for clinical use (telaprevir, boceprevir and simeprevir). These protease inhibitors (PIs) are potent inhibitors of HCV replication *in* *vivo*; however, resistance develops quickly even when these drugs are administered in combination with peginterferon/ribavirin. [1]–[3] Indeed, amino acid substitutions within the NS3 protease region have been identified *in* *vitro* and *in* *vivo* and correlated with reduced susceptibility to all PIs, including those in development (**Table 1**).

**Table 1:** List of known *in* *vitro* and *in* *vivo* amino acid substitutions resistant to NS3 protease inhibitors used in this study (NS3 residues 31 to 175).

NS3 aminoacid position	WT residue		Resistancesubstitutions	Protease Inhibitor*
	G1a	G1b		
36	V	V	A,I,L,M	Linear (Boceprevir/Telaprevir)
41	Q	Q	R	Linear (Boceprevir); Macrocyclic (Danoprevir/Simeprevir)
43	F	F	C,I,S,V	Linear (Boceprevir); Macrocyclic (Danoprevir/Simeprevir)
54	T	T	A,C,G,S	Linear (Boceprevir/Telaprevir)
55	V	V	A,I	Linear (Boceprevir)
80	Q,K	Q,L	H,K,R	Macrocyclic (Danoprevir/Simeprevir)
138	S	S	T	Macrocyclic (Danoprevir)
155	R	R	G,I,K,M,Q,S,T	Linear (Boceprevir/Telaprevir); Macrocyclic (Danoprevir/Simeprevir)
156	A	A	G,S,T,V	Linear (Boceprevir/Telaprevir); Macrocyclic (Danoprevir/Simeprevir)
158	V	V	I	Linear (Boceprevir)
168	D	D	A,E,G,H,I,N,T,Y,V	Macrocyclic (Simeprevir/Danoprevir)
170	I	V,I	A,F,T	Linear (Boceprevir)
175	L	M	L	Linear (Boceprevir)

*For conciseness, 2 representatives from each PI class were chosen [1]–[3], [35], [37], [50]–[53].

Several NS5B polymerase nucleoside analog inhibitors (NIs) have shown clinical efficacy in combination with peginterferon/ribavirin, and in interferon-free regimens. These NIs include sofosbuvir, now approved for the treatment of HCV infection [4] (GS7977, a phosphoramidate prodrug of 2′-deoxy-2′-α-fluoro-β-C-methyluridine-5′-monophosphate), mericitabine (RG7128, prodrug of β-D-2′-deoxy-2′-fluoro-2′-C-methyl cytidine), [5] and the guanosine-based NIs, now stopped for safety reasons (GS-938, IDX-184 and INX-189). Sofosbuvir and mericitabine treatment rarely select for resistant variants, identified as carrying substitutions S282T and/or L159F/L320F in NS5B. [6]–[9] There are also several investigational non-nucleoside NS5B inhibitors (NNIs) including ABT-072, ABT 333 and setrobuvir which bind to the Palm I allosteric domain, [10], [11] BMS-791325 [12] and BI207127 which bind to the Thumb I site [13], and VX-222 and filibuvir which bind to the Thumb II site. [14], [15] In contrast to NI-resistant NS5B variants, NNI-resistant variants emerge rapidly *in* *vitro* and *in* *vivo*. [11], [15]–[18] However, NNI-resistant variants that confer resistance to inhibitors that bind to one allosteric site do not confer cross-resistance to inhibitors that bind to a different allosteric site (**Table 2**).

**Table 2:** List of known *in* *vitro* and *in* *vivo* amino acid substitutions resistant to NS5B polymerase inhibitors used in this study (NS5B residues 244–496).

NS5B aminoacid position	WT residue		Resistancesubstitutions	Polymerase Inhibitor*
	G1a	G1b		
282	S	S	T	Nucleoside Inhibitor (Mericitabine, Sofosbuvir)
316	C	C,N	N,Y	NNI/Palm I (ABT-072/ABT-333, Setrobuvir, Tegobuvir)
320	L	L	I, F	Nucleoside Inhibitor (GS-938, Mericitabine)**
321	V	V	I	Nucleoside Inhibitor (GS-938)**
392	L	L	I	NNI/Palm I (ABT-072/ABT-333)
411	N	N	S	NNI/Palm I (ABT-072/ABT-333)
414	M	M	I,L,T,V	NNI/Palm I (ABT-072/ABT-333, Setrobuvir)
419	L	L	I,M	NNI/Thumb II (Filibuvir, VX-222)
421	A	A	V	NNI/Thumb I (BMS-791325)
422	R	R	K	NNI/Thumb II (Filibuvir, VX-222)
423	M	M	A,I,T,V	NNI/Thumb II (Filibuvir, VX-222)
426	M	M	A,T	NNI/Thumb II (Filibuvir)
445	C	C	F,Y	NNI/Palm I (Setrobuvir, Tegobuvir)
448	Y	Y	H, C	NNI/Palm I (ABT-072/ABT-333, Setrobuvir, Tegobuvir)
451	C	C	R	NNI/Palm I (Setrobuvir)
452	Y	Y	H	NNI/Palm I (Tegobuvir)
482	I	I	L,S,T	NNI/Thumb II (Filibuvir, VX-222)
494	V	V	A,I,T	NNI/Thumb II (Filibuvir)
495	P	P	A,L,S	NNI/Thumb I (BI 207127, BMS-791325)
496	P	P	A,S,T	NNI/Thumb I (Benzimidazole derivatives)

*[6], [9], [11], [15]–[18], [54], [55].

**L320F when in combination with L159F confers low level resistance to MCB [9], L320I or V321I when in combination with C223H confers low level resistance to GS-938 [54].

HCV NS5B RdRp, lacking a proof reading function, mis-incorporates nucleotides at a rate of 1 per 10 000 bases copied. [19] The low fidelity of replication is compounded by a high replication rate that can result in the production of up to 10^12^ virions per day. [20] As a result, HCV exists in any given patient as a diverse collection of closely related variants termed quasispecies. [21] Although the original definition of a quasispecies requires an effectively infinite population size, population geneticists have nonetheless found quasispecies theory to be useful for finite viral populations with high mutation rates, and have generally accepted the use of the term quasispecies when applied to HCV and several other viral infections [22].

Recent studies on protease inhibitors telaprevir and boceprevir have shown higher virological response rates, and lower breakthrough rates, in G1b-infected patients than in G1a-infected patients. [1], [2], [23], [24] Similar results have been described for patients enrolled in Palm I NNI-based interferon-free regimen. [25], [26] However, it is not known whether this corresponds to a different distribution of variants in G1a and G1b quasispecies in regards to the proportion that contain naturally occurring DAA-resistance mutations before treatment; it is also unknown what the possible implications for the response to DAA treatment could be.

In this study, we sought to investigate the overall genetic diversity and the potential presence of low-abundant DAA-resistant variants (<25% of the sequence reads) in the NS3 and NS5B genes of HCV G1a and G1b viruses in isolates from >200 DAA-naïve patients using ultradeep pyrosequencing (UDPS) methodology. We characterized, on a quantitative scale, the abundance (defined as the frequency of a particular amino acid substitution in an isolate’s quasispecies as low as 0.5%) and prevalence (defined as the proportion of patients which carries a particular amino acid substitution) of more than 30 established DAA-resistant substitutions and polymorphisms in the NS3 and NS5B regions. We also compared the genetic variability of G1a and G1b isolates to determine whether differences in variability might be responsible for the lower virological responses to HCV PIs and Palm I NNI -DAA-containing therapy in patients infected with G1a compared with G1b viruses.

## Materials and Methods

### Clinical isolates

Samples from 208 G1 DAA-naive patients, most of whom were enrolled in Roche-sponsored global clinical trials (PV18369, NV19865, PP22205, NP22660, NV21075 and PP25213, representing >90% of the isolates, remaining isolates being purchased from the American Red Cross) [27]–[32], with a median viral load of 3.6×10^6^ IU/mL (range 7.6×10^4^–5.9×10^7^ IU/mL) were studied. All studies were global and conducted in full conformance with the principles of the Declaration of Helsinki and Good Clinical Practice. Protocols and all amendments were reviewed and approved by local ethics committees and regulatory authorities. Written informed consent was obtained from all patients before any study-related activities occurred. The authors were not involved in any of the original sample collections and samples were de-identified prior to being accessed by the authors.

HCV RNA was extracted from 400 µl to 800 µl of serum from HCV-infected patients using the ZR Whole-blood Total RNA kit (Zymo Research, Irving, CA, USA) and following the manufacturer’s instructions.

### Population-based dideoxy-terminator (Sanger) sequencing

Population sequencing spanning the NS5B and NS3/4A coding regions was performed by Sanger sequencing using primers covering both DNA strands using an ABI 3730 xl DNA Analyzer. Chromatograms were analyzed using Sequencher (Gene Codes Corporation, Ann Arbor, MI, USA) and Vector NTI (Life Technologies, Carlsbad, CA, USA) software. Population sequence of each sample was then used for the 454 reads analysis (see section 454 sequence analysis).

### HCV amplification for 454 sequencing

Reverse transcription (RT) was performed using the Transcriptor High Fidelity cDNA Synthesis Kit (Roche Diagnostics, Indianapolis, IN, USA) following the manufacturer’s instructions, using random hexamers as primers for initiation and 1 to 9.15 µL of HCV RNA (depending on sample availability) per reaction. The RT cycling conditions were as follow: 5 minutes (min) at 25°C, 30 min at 50°C, 5 min at 85°C. For clinical isolates obtained from patients with an HCV viral load <3.4×10^5^ IU/mL, cDNAs were pooled (between 2 to 6 RT reactions, depending on sample availability) and concentrated using the Zymo Research DNA Clean & Concentrator-5 (Zymo Research, Irvine, CA, USA) following the manufacturer instruction.

Amplification of NS5B region (generating a 985 nucleotide fragment for G1a samples and a 916 nucleotide fragment for G1b samples) and NS3 region (generating a 750 nucleotide fragment for G1a samples and an 806 nucleotide fragment for G1b samples) was carried out in duplicate using the FastStart High Fidelity PCR System (Roche Diagnostics, Indianapolis, IN, USA) as follows: 4 µL cDNA (or 2 µl concentrated cDNA) was included in a 50 µL reaction mixture containing 5 µL Buffer #2 (1X final with 1.8 mM MgCl_2_), 1 µL dNTPs (0.2 mM final each), 1 µL each forward and reverse primers (0.4 µM final each) and 0.5 µL enzyme (2.5 Units). PCR reactions were performed as follow: 3 min at 94°C, then 30 cycles of 30 seconds (sec) at 94°C, 30 sec at 52°C, and 1 min at 72°C, followed by a final 7 min extension step at 72°C. The duplicate products of this first round PCR (PCR1) were pooled and then amplified in a nested PCR (PCR2) using 454-fusion primers containing custom 7-mer bar codes (designed to be able to pool amplified DNA from different patient samples for the UDPS reactions) and the same cycling conditions as PCR1. The PCR2 reaction produced 3 smaller overlapping amplicons (308–368 nucleotides) encompassing NS5B amino acids 244 to 496 and 2 smaller overlapping amplicons (244–356 nucleotides), encompassing NS3 amino acids 31 to 175 and 30 to 190 for G1a and G1b isolates, respectively.

The PCR2 products were double purified using AMPure beads (Beckman Coulter, Brea, CA, USA), quantified using Quant-iT PicoGreen dsDNA Reagent (Life Technologies, Carlsbad, CA, USA), pooled at equimolar concentrations and pyrosequenced using the 454/Roche GS FLX titanium platform producing reads of 331 bp on average. All amplification primers are given in **Tables S1 and S2 in File S1**.

### 454 sequence analysis

Standard flowgram format (SFF) files, the raw output from UDPS, were processed to generate paired files containing FASTA sequence reads and Phred-equivalent quality scores for each sequence library. To reduce sequence artifacts, reads shorter than 200 nucleotides and reads containing one or more bases with a quality score of <10 (>10% probability of sequence error) or a mean quality score <25 (>0.3% probability of sequence error per base per sequence read) were excluded. Sequenced reads were de-multiplexed using the 5′ primer and barcode sequences, resulting in the assignment of each read to a patient sample and primer-pair, and each UDPS read was aligned to the population-based sequence of each sample using the MosaikAligner program (http://bioinformatics.bc.edu). For the calling of nucleotide mutations and amino acid substitutions, each amplicon was compared to the corresponding subtype reference sequence, H77 for G1a (GenBank accession number M67463) and Con 1 for G1b (GenBank accession number AJ238799). High-abundance variants were defined as mutations present in ≥25% of the sequence reads (as is conventionally defined for the Sanger sequencing detection threshold), and low-abundance variants were defined as mutations present in <25% of the sequence reads.

All sequence data have been deposited at the NCBI Sequence Read Archive under study accession SRP040802.

The technical error rate was estimated for each UDPS run by amplifying and sequencing two HCV reference plasmids, G1a H77 and G1b Con1 using the same PCR protocol described for the clinical isolates. Each UDPS read was aligned to the corresponding plasmid sequence using the MosaikAligner program (http://bioinformatics.bc.edu) and the number of mismatches was counted. Homopolymeric regions were defined as regions with three or more identical consecutive nucleotides and their flanking nucleotides [33].

### Distribution of NS3 and NS5B quasispecies variants

The number and frequency of distinct virus variants (sometimes referred to as “haplotypes”) in each sample was estimated using the computational method ShoRAH (Short Reads Assemby into Haplotypes), which incorporates sequence error calculations to avoid overestimating the number of distinct genetic variants in a sample. [34] After removing virus variants present at frequency lower than 0.5%, the intra-sample quasispecies Shannon entropy, S_QS(nt)_, was calculated: [21].
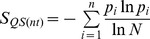
where p_i_ is the frequency of sequence variant i, n is the total number of variants and N is the average number of reads per amplicon. Each distinct variant identified by ShoRAH was translated to its corresponding amino acid sequences. Variants with same amino acid sequences were pooled and the Shannon entropy for the amino acid sequences (S_QS(aa)_) was calculated in a manner analogous to S_QS(nt)_. Shannon entropy was chosen to characterize the mutant spectra of viral quasispecies in our samples, because it is a simple normalized quantitative measure of variability (randomness) in a sequence dataset which incorporates both the frequency and the number of possible sequence variants into a single metric. In the context of quasispecies analysis, a higher Shannon entropy (e.g. 0.25) typically implies that a virus population consists of a large number of distinct sequence variants (e.g. ∼15 or more) occurring at low to moderate frequency, whereas a relatively low Shannon entropy (e.g. 0.02) often indicates a more ordered virus population which may contain just one to a few (e.g. ∼3) sequence variants occurring at significant frequency [21].

### Statistical inference

The Mann-Whitney-Wilcoxon test was used to assess the statistical significance of differences between the median Shannon entropy between subtypes and genes. The Fisher’s exact test was used to determine if the levels of amino-acid conservation in the NS3 and NS5B regions differed between G1a and G1b isolates by using the fisher.test function for count data, as implemented in the R statistical package.

### NS3 and NS5B drug-resistant amino acid substitutions included in the study

Study-defined NS3 and NS5B DAA-resistant amino acid substitutions are listed in Tables 1 and 2.

## Results

### UDPS coverage and technical error rate according to gene and genotype

UDPS yielded a total of 3.5 million high quality sequence reads of 200 or more nucleotides from 136 G1a and 55 G1b NS3 samples and from 77 G1a and 39 G1b NS5B samples. Overall, there was a median of 8,399 reads (IQR: 6,943–11,151) for the 191 NS3 samples and a median of 14,043 reads (IQR: 11347–16142) for the 116 NS5B samples. A total of 34 HCV plasmid controls were run (10 G1a and 8 G1b NS3 controls, and 10 G1a and 6 G1b NS5B controls) and a total of 339,769 sequenced reads with >200 bases were generated over 11 plates for the 34 controls: 105,210 for G1a NS3 and 80,462 for G1a NS5B; and 75,182 for G1b NS3 and 78,915 for G1b NS5B.

The median mismatch error rate (or technical error rate), determined using G1a H77 and G1b Con1 plasmid DNA, was 7.0×10^−4^ overall: 4.0×10^−4^ in non-homopolymeric regions and 1.4×10^−3^ in homopolymeric regions. The median mismatch error rate was the same for the NS3 and NS5B genes and for G1a and G1b plasmids. Assuming that errors occurred in a Poisson distribution and that samples contained one thousand viral templates, and adding the published error rate of the Roche Transcriptor High Fidelity reverse transcriptase (1.98×10^−5^), the likelihood that technical artefact would cause a mutation to be detected at a level of 0.5% or higher would be 7.7×10^−5^ in non-homopolymeric regions and 1.5×10^−2^ in homopolymeric regions. The likelihood that technical artefact could cause a mutation to be detected at a level of 1.0% or higher would be <1.0×10^−10^ in non-homopolymeric regions and 2.5×10^−6^ in homopolymeric regions. Using the overall median error rate of 7.2×10^−4^ and a Bonferroni correction for testing 13 PI-resistant variants, 3 NI-resistant variants, and 18-NNI-resistant variants, the likelihood of detecting any PI-, NI-, or NNI-resistance mutation at a level of 0.5% or higher in a sample would be 1.2×10^−2^, 2.7×10^−3^, and 1.6×10^−2^, respectively. To prevent false positives, a conservative cut-off (≥0.5%) for variant detection was adopted as the minimum threshold in calling high- and low-abundance mutations.

### Distributions of quasispecies diversity among G1a and G1b isolates

**Figure 1:**
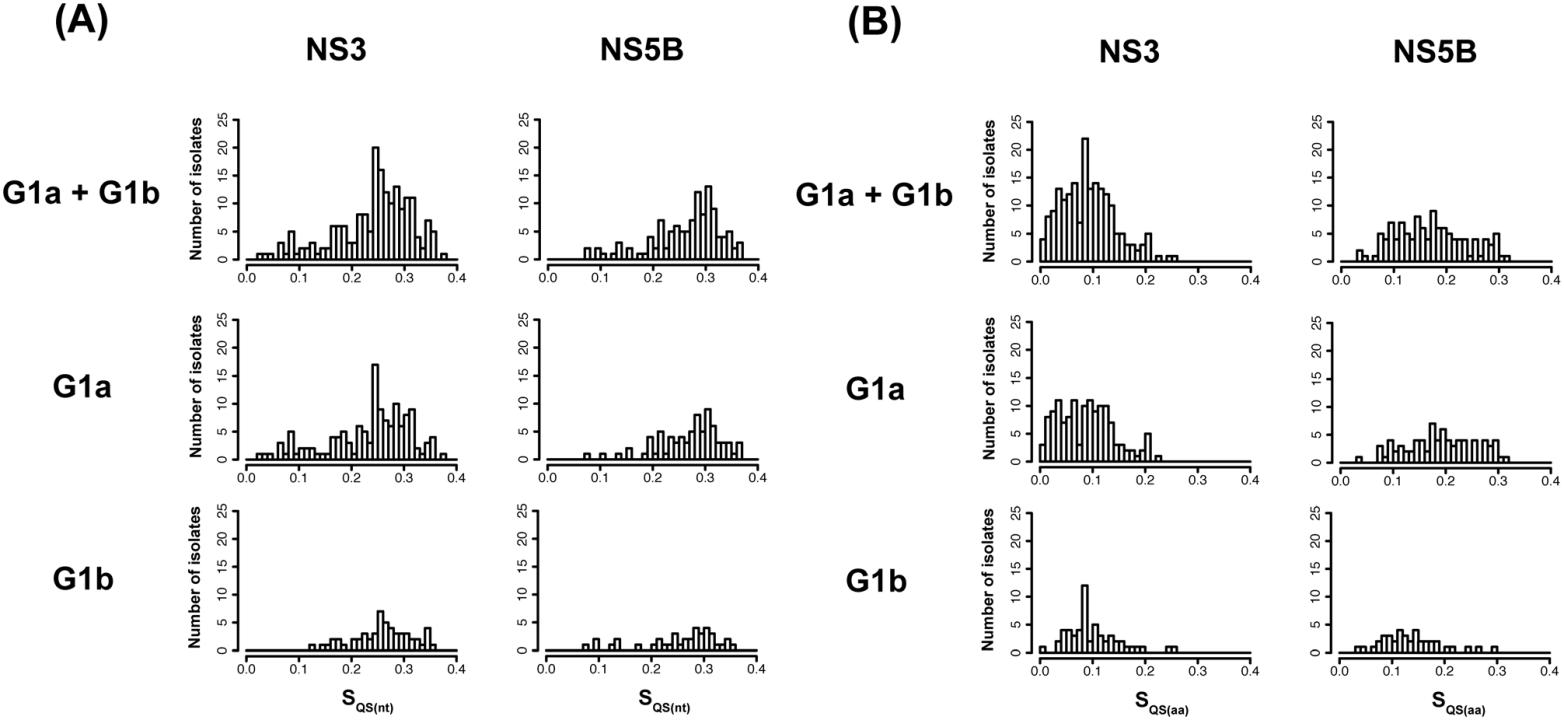
Histograms of baseline quasispecies diversity in the study population, as represented by Shannon entropy in nucleotides (A) and in amino acids (B). Sequence variants were assembled using ShoRAH (as described in material and methods) and only variants with frequencies ≥0.5% were included.

To evaluate inter-patient genetic variation across the NS3 and NS5B quasispecies obtained by UDPS, the histograms of Shannon entropy of quasispecies (see Materials and Methods for definition) were plotted for all samples combined (G1a and G1b), or separately by subtype, in nucleotides (**Figure 1A**) and in amino acids (**Figure 1B**).

The median Shannon entropy for the NS3 nucleotide sequences was similar for the 136 G1a (0.25, Inter Quantile Range [IQR]: 0.19 to 0.29) and 55 G1b (0.26. IQR: 0.23 to 0.29) samples (p = 0.1; Mann Whitney Test) (**Figure 1A**). The median Shannon entropy for the NS5B nucleotide sequences was similar for the 77 G1a (0.28, IQR: 0.23 to 0.31) and 39 G1b (0.28, IQR: 0.22 to 0.31) samples (p = 0.3; Mann Whitney Test) (**Figure 1A**).

The median Shannon entropy for the translated NS3 sequences was similar for the 136 G1a (0.09, IQR: 0.04 to 0.12) and 55 G1b (0.09, IQR: 0.06 to 0.13) samples (p = 0.2) (**Figure 1B**) but it was higher for the translated NS5B sequences for the 77 G1a (0.19, IQR: 0.14 to 0.24) compared with the 39 G1b (0.13, IQR: 0.09 to 0.17) samples (p<0.001) (**Figure 1B**).

The amino acid sequences showed less variability (i.e. lower Shannon entropy) than the nucleotide sequences (S_QS(aa)_: ∼0 to 0.24 vs S_QS(nt)_: ∼0 to 0.31, zero-th to 75th percentile, **Figures 1A & B**). Both G1a and G1b virus populations exhibit Shannon entropy ranges spanning S∼0 and S∼0.3 (**Figures 1A & B**), consistent with the notion that sequence diversity in HCV patient samples can vary among samples.

### Prevalence of high-abundance NS3 resistance amino acid substitutions (present in ≥25% of UDPS reads)

Among the 136 G1a NS3 isolates, 48 (33%) of the 145 sequenced residues had one or more high-abundance amino acid substitutions, whereas the remaining 97 (67%) sequenced residues had no high-abundance variants (**Table 3**). Among the 55 G1b NS3 isolates, 33 (23%) of the 145 sequenced residues had one or more high-abundance amino acid substitutions, whereas the remaining 112 (77%) of sequenced residues had no high-abundance variants (**Table 3**).

**Table 3:** Amino acid diversity in the NS3 protease region of 136 G1a and 55 G1b isolates from treatment-naïve HCV infected subjects.

GT 1a isolates				GT 1b isolates			
AA	Amino acid(≥25%abundance)	AA	Amino acid(≥25%abundance)	AA	Amino acid(≥25%abundance)	AA	Amino acid(≥25%abundance)
33	**V** ^127^ I^9^	87	**A** ^134^ V^1^ S^1^	33	**V** ^53^ I^2^	122	**S** ^51^ G^3^ T^1^
35	**I** ^134^ V^2^	88	**P** ^135^ L^1^	46	**T** ^50^ S^4^ A^1^	130	**R** ^54^ K^1^
36	**V** ^135^ M^1^	89	**Q** ^124^ P^9^ H^2^ S^1^	48	**V** ^36^ I^19^	132	**V** ^40^ I^15^
39	**A** ^131^ T^5^	91	**A** ^102^ S^21^ T^12^ C^1^	49	**N** ^54^ S^1^	146	**P** ^54^ S^1^
40	**A** ^94^ T^40^ V^1^ S^1^	94	**L** ^128^ M^8^	50	**G** ^54^ N^1^	147	**S** ^50^ L^4^ M^1^
42	**T** ^134^ S^2^	95	**T** ^135^ A^1^	51	**V** ^52^ A^2^ T^1^	150	**A** ^53^ V^2^
46	**T** ^132^ S^4^	98	**T** ^131^ A^3^ S^2^	54	**T** ^54^ S^1^	153	**I** ^52^ V^3^
48	**I** ^135^ V^1^	107	**V** ^135^ I^1^	56	**Y** ^41^ F^14^	170	**V** ^40^ I^15^
49	**N** ^135^ G^1^	110	**H** ^134^ Q^2^	61	**S** ^48^ A^4^ T^1^ P^2^	174	**S** ^53^ A^1^ T^1^
51	**V** ^131^ A^4^ T^1^	113	**V** ^134^ I^2^	67	**P** ^52^ Q^2^ A^1^		
52	**C** ^135^ M^1^	114	**I** ^134^ V^2^	68	**K** ^53^ T^2^		
54	**T** ^132^ S^4^	116	**V** ^134^ I^2^	71	**I** ^43^ V^12^		
55	**V** ^131^ I^3^ A^2^	122	**S** ^127^ G^7^ N^1^ C^1^	72	**T** ^36^ I^13^ A^3^ S^1^ V^2^		
61	**T** ^126^ S^6^ A^4^	124	**G** ^135^ A^1^	80	**Q** ^54^ L^1^		
62	**R** ^130^ K^6^	127	**L** ^135^ I^1^	86	**Q** ^44^ P^9^ L^2^		
64	**I** ^112^ L^23^ M^1^	146	**P** ^134^ S^2^	89	**P** ^46^ S^9^		
66	**S** ^130^ T^4^ A^2^	147	**A** ^132^ S^2^ V^1^ M^1^	94	**L** ^53^ M^2^		
67	**P** ^118^ S^17^ A^1^	150	**A** ^132^ V^4^	95	**T** ^54^ A^1^		
68	**K** ^130^ T^3^ N^2^ R^1^	151	**V** ^133^ A^1^ T^2^	101	**S** ^54^ N^1^		
71	**V** ^132^ I^1^ A^3^	153	**I** ^133^ L^3^	105	**Y** ^54^ F^1^		
72	**I** ^135^ V^1^	168	**D** ^135^ E^1^	108	**T** ^54^ S^1^		
80	**Q** ^102^ K^30^ R^3^ L^1^	170	**I** ^128^ V^8^	110	**H** ^53^ Y^2^		
83	**V** ^127^ I^9^	174	**N** ^76^ S^59^ A^1^	114	**I** ^54^ V^1^		
86	**P** ^131^ A^5^	175	**L** ^135^ M^1^	117	**R** ^54^ H^1^		

Superscript numbers represent the number of isolates with the particular noted residue. Amino acid representing the wild type is indicated in bold. Residues known for resistance substitutions are underlined.

The polymorphic PI-resistant substitution Q80K, which is associated with reduced low-level susceptibility to the macrocyclic PIs simeprevir and danoprevir, was present in high abundance (in 30 to 100% of the reads) in 30 (22%) of the 136 G1a isolates but in none of the 55 G1b isolates (p<0.001; Fisher’s Exact Test). Additional high-abundance PI-resistant amino acid substitutions in G1a samples were found (in 41% to 99.8% of the reads), including V36M (n = 1), T54S (n = 4), V55A (n = 2) and V55I (n = 3), which confer resistance to linear PIs, and Q80R (n = 3) and D168E (n = 1), which confer resistance to macrocyclic PIs. One G1b sample contained the PI-resistant variant T54S (n = 1, in 99.6% of the reads). Overall (including Q80K), G1a isolates (44/136; 32%) were more likely to contain one or more high abundance amino acid substitutions than were G1b isolates (1/55, 2%; p<0.001; Fisher’s Exact Test). Excluding Q80K, the difference in the proportions of high-abundance amino acid substitutions between G1a (14/136, 10.3%) and G1b (1/55; p = 0.07, Fisher’s Exact Test) was smaller. In all but one G1a isolates, a Leucine residue was present in NS3 position 175. This amino acid variant was not included in our analysis of potential resistance-associated variants, because it has only been associated with resistance to boceprevir in the G1b context.

### Prevalence of low-abundance NS3 resistance amino acid substitutions (present in <25% of UDPS reads)

Among the 136 NS3 G1a isolates, 20 (15%) had one or more low-abundance PI-resistant amino acid substitutions (in 0.5% to 15.2% of the reads) associated with reduced susceptibility to PIs: V36A (n = 1), V55A (n = 2), V55I (n = 1), Q80K (n = 2), Q80R (n = 4), R155K (n = 1), A156G (n = 1), A156T (n = 1), V158I (n = 1), D168N (n = 1), D168V (n = 1), I170T (n = 2) and dual amino acid substitutions Q80R/I170T (n = 1) and R155K/D168G (n = 1) (**Table 4**).

**Table 4:** Low-abundance NS3 PI-resistant amino acid substitutions found in 20/136 G1a and in 5/55 G1b treatment-naïve HCV samples.

Sample ID	HCV Genotype	Low-abundance PIresistance^1^	Class of Proteaseinhibitor
Pt 4	1a	R155K (0.6% = 14/2301)	Linear and Macrocyclic
Pt 7	1a	A156T (0.8% = 20/2281)	Linear and Macrocyclic
Pt 40	1a	I170T (0.6% = 17/2575)	Linear
Pt 42	1a	Q80K (15.1% = 8490/55864)	Macrocyclic
Pt 50	1a	Q80R (6.7% = 826/12154)	Macrocyclic
Pt 52	1a	D168V (0.9% = 48/5047)	Macrocyclic
Pt 54	1a	V55A (0.5% = 15/2993)	Linear
Pt 59	1a	V55I (15.2% = 543/3559)	Linear
Pt 60	1a	V55A (0.5% = 24/4664)	Linear
Pt 63	1a	Q80R (0.6% = 43/6945)	Macrocyclic
Pt 65	1a	A156G (3.5% = 123/3512)	Linear and Macrocyclic
Pt 67	1a	D168N (3.8% = 170/4390)	Macrocyclic
Pt 68	1a	V36A (1.3% = 39/2795)	Linear
Pt 70	1a	I170T (0.8% = 51/6061)	Linear
Pt 71	1a	V158I (0.5% = 26/4813)	Linear
Pt 73	1a	Q80R (1.7% = 331/18914)	Macrocyclic
Pt 74	1a	Q80K (1% = 85/8202)	Macrocyclic
Pt 76	1a	Q80R (0.5% = 59/11582)	Macrocyclic
Pt 77	1a	Q80R (0.8% = 189/21037),I170T (0.7% = 62/8663)	Linear and Macrocyclic
Pt 81	1a	R155K (0.5% = 31/5635),D168G (0.5% = 30/5612)	Linear and Macrocyclic
Pt 33	1b	D168E (2.7% = 129/4688),M175L (3.7% = 176/4687)	Linear and Macrocyclic
Pt 82	1b	V36A (0.6% = 13/2133)	Linear
Pt 83	1b	V170T (1.3% = 34/2504)	Linear
Pt 84	1b	M175L (0.6% = 48/6974)	Linear
Pt 85	1b	V170A (0.7% = 12/1683)	Linear

1 In parenthesis, abundance of the variant is indicated (in % and number of reads in which the variant is detected over the number of total reads).

Among the 55 G1b isolates, five (9%) contained low-abundance PI-resistant amino acid substitutions (in 0.6% to 3.7% of reads) associated with reduced susceptibility to PIs: V36A (n = 1), V170A (n = 1), V170T (n = 1), and M175L (n = 1) and dual amino acid substitutions D168E/M175L (n = 1) (**Table 4**). There was no significant difference in the prevalence of low-abundance PI-resistant amino acid substitutions between G1a (15%, 20/136) and G1b isolates (9%, 5/55; p = 0.4, Fisher’s Exact Test).

Low-abundance variants at amino acid positions associated with PI resistance, but containing amino acid substitutions not previously described as conferring resistance to PIs, were present at low prevalence (Q41H (n = 2), F43L (n = 7), V55N (n = 1), Q80L (n = 5), S138P (n = 5), V158A/G/M (n = 3), in G1a isolates; and F43L (n = 1), Q80L (n = 5), V170M (n = 1) in G1b isolates) (data not shown).

### Prevalence of high-abundance NS5B resistance amino acid substitutions (present in ≥25% of UDPS reads)

Amino acid substitutions in NS5B detected in ≥25% of UDPS reads after translation of the 77 G1a and 39 G1b isolates are listed in Table 5. Of the 253 sequenced NS5B amino acid residues, 179 (71%) did not show a substitution present in ≥25% of the UDPS reads in any of the isolates. The remaining 74 NS5B amino acid residues (29%) had one or more substitution present in ≥25% of the UDPS reads (**Table 5**). Among them, NNI-resistance amino acid substitutions were present in 21 G1a isolates (M414V [n = 1] and C445F [n = 1] in the Palm I domain, A421V [n = 15] in the Thumb I domain, I482L [n = 1] in the Thumb II domain and dual amino acid substitutions A421V/M423I [n = 2] and A421V/M423V [n = 1]) and 10 G1b isolates (C316N [n = 3], M414L [n = 1], M414T [n = 1] and dual amino acid substitutions C316N/M414L [n = 1] in Palm I domain and A421V [n = 3] and P496A [n = 1] in the Thumb I domain), all present in 72.2% to 100% of the reads. No NI-resistant variants were detected. There was no significant difference in the prevalence of high-abundance NNI-resistant variants between subtypes G1a and G1b (21/77 vs 10/39; p>0.1, Fisher’s Exact test).

**Table 5:** Amino acid diversity in the NS5B polymerase region of 77 G1a and 39 G1b isolates from treatment-naïve HCV infected subjects.

GT 1a isolates				GT 1b isolates			
AA	Amino acid(≥25%abundance)	AA	Amino acid(≥25%abundance)	AA	Amino acid(≥25%abundance)	AA	Amino acid(≥25%abundance)
251	**V** ^75^ L^1^ M^1^	405	**V** ^75^ I^2^	246	**A** ^38^ P^1^	393	**A** ^38^ V^1^
254	**K** ^70^ R^7^	412	**I** ^75^ V^2^	250	**R** ^36^ K^3^	399	**T** ^38^ S^1^
255	**S** ^76^ A^1^	414	**M** ^76^ V^1^	251	**Q** ^37^ R^1^ K^1^	401	**R** ^34^ K^5^
267	**T** ^76^ F^1^	415	**F** ^75^ Y^2^	252	**A** ^37^ V^2^	412	**I** ^38^ V^1^
270	**R** ^73^ K^4^	421	**A** ^60^ V^17^	254	**R** ^26^ K ^13^	414	**M** ^36^ L^2^ T^1^
297	**I^7^** ^6^ L^1^	423	**M** ^74^ I^2^ V^1^	262	**I** ^35^ V^4^	415	**Y** ^38^ F^1^
300	**Q** ^50^ R^22^ L^3^ K^2^	424	**I** ^71^ V^6^	266	**L** ^38^ M^1^	421	**A** ^36^ V^3^
307	**G** ^75^ R^1^ K^1^	425	**L** ^75^ F^1^ M^1^	273	**N** ^38^ T^1^	424	**I** ^36^ V^3^
309	**R** ^42^ Q^35^	426	**M** ^67^ L^10^	300	**S** ^28^ T^8^ A^3^	425	**L** ^38^ M^1^
310	**D** ^70^ N^6^ A^1^	431	**S** ^75^ G^2^	309	**Q** ^35^ R^4^	426	**M** ^38^ L^1^
327	**A** ^40^ Q^31^ V^5^ R^1^	432	**V** ^71^ I^6^	310	**C** ^38^ S^1^	432	**I** ^38^ V^1^
330	**Q** ^73^ P^3,^ E^1^	434	**I** ^71^ M^3^ L^3^	311	**C** ^38^ Y^1^	435	**A** ^38^ V^1^
334	**A** ^76^ E^1^	435	**A** ^76^ T^1^	316	**C** ^35^ N^4^	440	**E** ^35^ G^4^
335	**S** ^74^ N^2,^ G^1^	436	**R** ^76^ K^1^	327	**A** ^38^ E^1^	441	**K** ^37^ Q^1^ R^1^
337	**R** ^75^ K^2^	445	**C** ^76^ F^1^	329	**T** ^38^ I^1^	442	**A** ^38^ T^1^
341	**E** ^76^ V^1^	459	**L** ^ 76^ I^1^	333	**A** ^37^ E^2^	443	**L** ^38^ I^1^
355	**Q** ^ 76^ H^1^	461	**P** ^ 73^ L^4^	335	**S** ^35^ N^4^	444	**C** ^38^ E^1^
362	**L** ^75^ R^1^ P^1^	464	**Q** ^76^ E^1^	338	**V** ^37^ A^2^	446	**Q** ^38^ E^1^
376	**G** ^71^ S^3^ D^2^ N^1^	473	**S** ^76^ T^1^	353	**P** ^38^ L^1^	461	**Q** ^35^ P^3^ L^1^
377	**A** ^66^ T^11^	480	**G** ^73^ S^4^	355	**Q** ^31^ K^8^	463	**I** ^38^ V^1^
379	**K** ^75^ R^2^	482	**I** ^76^ L^1^	374	**H** ^38^ F^1^	464	**Q** ^34^ E^5^
389	**T** ^76^ L^1^	483	**N** ^76^ T^1^	376	**A** ^38^ G^1^	476	**S** ^38^ T^1^
392	**L** ^73^ F^4^	487	**A** ^73^ T^4^	377	**S** ^36^ T^2^ N^1^	480	**G** ^37^ A^1^ S^1^
399	**T** ^76,^ S^1^			381	**V** ^38^ T^1^	487	**S** ^35^ A^4^
401	**R** ^75,^ K^2^			392	**L** ^38^ I^1^	496	**P** ^38^ A^1^

Numbers in superscript represent the number of isolates with a particular noted residue. Amino acid representing the wild type is indicated in bold. Residues known for resistance substitutions are underlined. Note: For residue 426, the M426L has not been described as a mutation conferring resistance (see Table 2).

### Prevalence of low-abundance NS5B resistance amino acid substitutions (present in <25% of UDPS reads)

Low-abundance NNI-resistant variants were present in 29 (38%) of the 77 G1a and 12 (31%) of the 39 G1b samples (p = 0.5; Fisher’s Exact test). The 29 G1a samples contained the following NNI-resistant amino acid substitutions in 0.5% to 20% of reads: C316Y (n = 1), M414T (n = 1), M414V (n = 1), C445Y (n = 1), Y448C (n = 1), Y448H (n = 4), Y452H (n = 1) and dual amino acid substitutions M414T/M414V (n = 1) in the Palm I domain; A421V (n = 10) in the Thumb I domain; M423I/I482T (n = 1), V494A (n = 2), V494I (n = 1) in the Thumb II domain, and dual amino acid substitutions of C316Y/A421V (n = 1), M414T/V494A (n = 1), A421V/M426T (n = 1), M423T/Y452H (n = 1) (**Table 6**). The GS-938-NI-resistant variant V321I was present in 0.7% of reads of one G1a sample.

**Table 6:** Low-abundance NS5B drug-resistant variants found in 30/77 G1a and in 12/39 G1b treatment-naïve HCV samples.

Sample ID	HCV GT	Low-abundanceNI and NNI resistance^1^	Class of Polymeraseinhibitor
Pt 1	1a	M414T (4.2% = 169/3979)	Palm I
Pt 2	1a	C316Y (0.8% = 53/6455)	Palm I
Pt 3	1a	Y448H (0.6% = 18/2607)	Palm I
Pt 4	1a	V494A (2.6% = 96/3691)	Thumb II
Pt 5	1a	M414T (0.6% = 19/2781),M414V (0.6% = 17/2781)	Palm I
Pt 6	1a	C316Y (0.6% = 52/7859),A421V (1.4% = 34/2406)	Palm I; Thumb I
Pt 7	1a	M414T (0.5% = 13/2595),V494A (1.9% = 47/2381)	Palm I; Thumb II
Pt 8	1a	Y448H (1.1% = 32/2669)	Palm I
Pt 9	1a	M423I (0.5% = 13/2277),I482T (0.9% = 24/2564),	Thumb II
Pt 10	1a	A421V (1.8% = 78/4256),M426T (0.6% = 28/4296)	Thumb I; Thumb II
Pt 11	1a	M423T (1.2% = 93/7606),Y452H (0.8% = 72/8416)	Thumb II; Palm I
Pt 12	1a	C445Y (2.9% = 89/3013)	Palm I
Pt 15	1a	V321I (0.7% = 56/7773)	Nucleoside Inhibitor
Pt 17	1a	Y448H (0.7% = 42/5676)	Palm I
Pt 18	1a	Y452H (0.5% = 16/3012)	Palm I
Pt 19	1a	Y448H (1.1% = 38/3402)	Palm I
Pt 20	1a	V494A (1.7% = 74/4258)	Thumb II
Pt 21	1a	Y448C (0.5% = 31/5402)	Palm I
Pt 39	1a	A421V (1.6% = 108/6516)	Thumb I
Pt 44	1a	A421V (0.7% = 20/2704)	Thumb I
Pt 46	1a	A421V (2.3% = 53/2281)	Thumb I
Pt 51	1a	V494I (20% = 378/1896)	Thumb II
Pt 52	1a	A421V (0.7% = 36/4661)	Thumb I
Pt 57	1a	M414V (2% = 116/5626)	Palm I
Pt 59	1a	A421V (1.5% = 51/3203)	Thumb I
Pt 93	1a	A421V (4.3% = 220/5009)	Thumb I
Pt 99	1a	A421V (1.2% = 23/1873)	Thumb I
Pt 118	1a	A421V (1.4% = 33/2224)	Thumb I
Pt 129	1a	A421V (0.7% = 28/3510)	Thumb I
Pt 142	1a	A421V (0.9% = 30/3117)	Thumb I
Pt 23	1b	Y452H (14.3% = 912/6346)	Palm I
Pt 26	1b	Y452H (21.3% = 897/4202)	Palm I
Pt 27	1b	Y452H (0.7% = 43/5376)	Palm I
Pt 29	1b	M426T (0.6% = 35/5167)	Thumb II
Pt 31	1b	P496S (1.8% = 75/3974)	Thumb I
Pt 32	1b	Y448C (0.7% = 43/5681)	Palm I
Pt 33	1b	M414I (0.7% = 24/3045),I482L (4.5% = 150/3275),	Palm I; Thumb II
Pt 34	1b	A421V (0.6% = 34/4890)	Thumb I
Pt 35	1b	A421V (0.5% = 31/5317),Y448H (0.7% = 38/5426)	Thumb I; Palm I
Pt 37	1b	Y452H (1.4% = 75/5059)	Palm I
Pt 38	1b	Y448H (0.7% = 20/2629),V494I (0.6% = 14/2191)	Palm I; Thumb II
Pt 161	1b	V494I (1.1% = 42/3793)	Thumb II

1 In parenthesis, abundance of the variant is indicated (in % and number of reads in which the mutation is detected over the number of total reads).

The 12 G1b samples contained the following NNI-resistant amino acid substitutions in 0.6% to 21.3% of reads: Y448C (n = 1), Y452H (n = 4) in the Palm I domain; A421V (n = 1) and P496S (n = 1) in the Thumb I domain; M426T (n = 1), V494I (n = 1) in the Thumb II domain and dual amino acid substitutions of M414I/I482L (n = 1), A421V/Y448H (n = 1), Y448H/V494I (n = 1) (**Table 6**).

The NI-resistant variants S282T and L320I/F were not present in any read from G1a or G1b samples.

### Most natural DAA-resistant variants detected involved the lowest genetic barrier (single transition)

Overall, 75% (18/24) of the NI- and NNI-resistant amino acid substitutions detected in G1a or G1b isolates were attributed to a single transition, while 21% (5/24) were the results of a single transversion (**Table 7**). No observed NS5B substitution represented a double transversion from wild type. Only 4% (1/24) involved 1 transition and 1 transversion (C316N). Similarly, 69% (13/19) of the PI-resistant amino acid substitutions detected in G1a or G1b isolates were caused by a single transition whereas 26% (5/19) were due to a single transversion, and only 5% (1/19) involved two transitions (**Table 7**).

**Table 7:** Contribution of each mutational category (transition and transversion) to the development of drug-resistant substitutions (variants) in the NS3 and NS5B proteins reported in this study.

	NS5B		NS3	
	G1a	G1b	G1a	G1b
**Single Transition**(GBS = 1)	C316Y (2)	M414I/T (1/1)	V36A/M (1/1)	V36A (1)
	V321I (1)	A421V (5)	V55A/I (4/4)	V170A (1)
	M414T/V (3/3)	M426T (1)	Q80R (8)	M175L (2)
	A421V (29)	Y448C/H (1/2)	R155K (2)	
	M423I/T/V (3/1/1)	Y452H (4)	A156T (1)	
	M426T (1)	V494I (2)	V158I (1)	
	C445Y (1)	P496S (1)	D168G/N (1/1)	
	Y448C/H (1/4)		I170T (3)	
	Y452H (2)			
	I482T (1)			
	V494A/I (3/1)			
**Double Transitions**(GBS = 2)				V170T (1)
**Single transversion**(GBS = 2.5)	C445F (1)	L392I (1)	T54S (4)	T54S (1)
	I482L (1)	M414L (2)	*Q80K (32)*	D168E (1)
		I482L (1)	A156G (1)	
		P496A (1)	D168E/V (1/1)	
**1 transition +** **1 transversion**(GBS = 3.5)		*C316N (4)*		

The genetic barrier score (GBS) was calculated according to the model described by Van de Vijver et al.(50) A score of 1 is assigned to transitions (A↔G and C↔T) and 2.5 to transversions (A↔C, A↔T, G↔C and G↔T). Natural polymorphisms are indicated in italic. Numbers of isolates are indicated in parenthesis.

## Discussion

Resistance profiles of the first four approved DAAs agents (the protease inhibitors telaprevir, boceprevir and simeprevir and NS5B polymerase inhibitor Sofosbuvir) have been well characterized and clinical resistance resulting in treatment failure has been reported for the first three. [1]–[3] It has recently been shown that virological response rates are higher and breakthrough rates are lower in G1b infected patients than in G1a infected patients treated with certain classes of DAAs such as PIs and Palm I NNIs [1], [2], [23]–[26].

As a result of a lack of a proof reading function in the HCV polymerase resulting in a high rate of spontaneous mutation, variants emerge constantly and, with a DAA selection pressure combined with a replicative advantage, may quickly become dominant in the population. It is not known if a higher level of genetic variability exists in G1a than G1b, or if elevated variability may be associated with treatment response to DAA’s. A thorough characterization of the genetic diversity and prevalence of DAA-resistant variants in treatment-naive HCV infected patients will therefore shed light on their potential clinical relevance and impact.

In this study, we tested the hypothesis of whether intrinsic genetic variability across G1 subtypes is directly associated with the differential response rates between G1a- and G1b-infected patients treated with DAAs. For this, we performed an extensive UDPS analysis that included 191 NS3 and 116 NS5B isolates from 208 HCV-infected DAA-naïve patients infected by a G1a or G1b virus with viral load of the patients spanning almost three orders of magnitude (see Materials and Methods). To our knowledge, this is the largest UDPS study on HCV diversity in treatment-naïve patients up to now.

We observed a higher prevalence of high-abundance variants containing a PI-resistant variant/polymorphism in G1a than G1b isolates. However, this higher prevalence is mainly driven by a polymorphism at position 80 that, in the case of simeprevir, pre-existence of Q80K polymorphism has been shown to affect the sustained virologic response (SVR) rate. [3] Amino acid substitution at Q80 have not been identified in telaprevir or boceprevir clinical trials [1], [2] and have no significant effects on the activity of telaprevir or boceprevir in *in* *vitro* experiments. [35] Low-abundance PI-resistant variants were also identified in both G1a and G1b isolates, however no significant difference between G1a and G1b was observed. The resistance mutations V36M and R155K in G1b isolates and V170A in G1a isolates have higher genetic barriers and require two nucleotide changes from wild-type (Table 7). Consistent with this phenomenon, we found no pre-existing V36M and R155K mutations among G1b isolates and no V170A mutations among G1a isolates in the set of high- and low-abundance mutations from this study.

The impact of low-level pre-existing PI-resistant variants on treatment outcome is yet to be fully determined. Recently published studies using UDPS methods and following a small number of PI-treated patients have shown that, in some (but not all) patients, pre-existing low-level PI-resistant variants could have affected their response to treatment [36] or re-treatment [37]; the presence of PI-resistant variants at baseline did not necessarily prevent a patient from responding to treatment and PI-resistant variants at baseline were not selectively enriched upon treatment. [29] Our findings support the notion that the relationships between pre-existing PI-resistant variants and treatment outcome are likely to be complex and may depend on virus genotype, genetic barriers of a particular resistant variant as well as the prevalence of the variant, among other factors.

In contrast to the NS3 region, no significant difference between G1a and G1b isolates was observed in the prevalence of high-abundance drug-resistant variants in the allosteric binding sites of the NS5B region. The results obtained here are also in agreement with previous studies that showed an overall 10 to 20% prevalence of variants at known drug resistance sites in the NS5B sequences from treatment-naive individuals. [38], [39] Low-abundance drug-resistant variants in the allosteric binding sites of the NS5B region were detected in ∼20 to 30% of the isolates, with no difference between G1a and G1b isolates.

The impact of NNI resistance variants abundance on drug susceptibility was demonstrated in an *in* *vitro* phenotypic study, showing that NNI-resistance variants present >25% decrease significantly the NNI potency. On the other hand, NNI-resistance variants present<25% did not necessarily have an impact on NNI potency [38].

This study also looked at the potential pre-existence of Sofosbuvir or Mericitabine resistance mutations. The NS5B nucleos(t)ide inhibitor-resistant variant S282T, was not found. To date, no S282T variant, has been found in treatment-naive GT 1 isolates above the assay detection limit of sensitive sequencing technology such as UDPS, as shown in this study (n = 116) and in a recent study. [40] A novel combination of NS5B substitutions (L159F/L320F) conferring a low-level resistance to mericitabine has been described recently. [9] Variants with L320F substitution were not observed, corroborating the absence of resistance to these compounds at low level in the treatment-naïve individuals included in this study. It is noteworthy that the only NS5B NI-resistant substitution detected in this study, V321I, was low-abundance and involves a low genetic barrier of a single transition. On the other hand, the NI-resistant substitution S282T and the NI-resistant combination L159F/L320F, which were not observed, have higher genetic barriers and require a transversion and two nucleotide changes, respectively. For the NS5B allosteric inhibitors, the observed resistance variants were single transitions, single transversions or a combination of single transition and single transversion (**Table 7**), with the majority being single transitions.

The quasispecies diversity of each isolate in each region and the inter-patient variation was also determined by the Shannon entropy at the nucleotide and amino acid levels. The only difference was seen between G1a and G1b isolates when comparing the Shannon entropy calculated for the NS5B amino acid sequences (0.19 vs 0.13; p<0.001; Mann-Whitney Test). When comparing the Shannon entropy between two patient groups, a statistical difference at the amino acid level was not always found to be correlated with a statistical difference at the nucleotide level (or vice versa), possibly due to codon degeneracy and other factors. For example, in a retrospective investigation of ten patients chronically infected with HCV G3a and treated with peginterferon/ribavirin, Moreau *et al* reported that, at the baseline time point, the treatment failure group was found to have a higher Shannon entropy at the amino acid level (but not at the nucleotide level) than the sustained virological responders group [41].

In summary, no significant difference in median S_QS(nt)_ levels was observed in this study that could differentiate G1a and G1b quasispecies in either the NS3 or NS5B region. Similar results have been reported in a recent smaller UDPS study, based on 8 G1a and 6 G1b samples. [36] It will be of future interest to investigate whether correlations can be established between Shannon entropy within the NS5B region at the amino acid level and treatment failure rates across clinical trials of G1a and G1b patients treated with NS5B NIs or NNIs.

Despite the high genetic variability of HCV, only a third of the amino acid positions in NS3 (∼23–33%) and NS5B (∼29%) were found to contain high-abundance substitutions among all isolates in this study. This apparent discrepancy can be attributed, at least partly, to the fact that synonymous substitution rates in HCV are typically >10-fold higher than the non-synonymous substitution rates in the NS3 and NS5 regions [42], leading to many more nucleotide changes which do not alter amino acid identities. Additional constraints, such as the requirement to preserve the 3D structures of proteins and essential base-pairing in RNA, may have further reduced the number of “neutral” sites, where sequence change can be tolerated with no significant effects on virus fitness.

High-throughput sequencing technology such as UDPS and Illumina deep sequencing has provided powerful new tools to characterize the genomes of pathogens such as HCV, and has been increasingly utilized to detect and quantify rare variants, which are below the detection limits of Sanger-based techniques such as clonal sequencing [36], [43]–[45]. However, the sensitive detection of rare variants requires the differentiation between actual variants and technical errors originating from library preparation and sequencing processes. By sequencing the NS3 and NS5B regions of two HCV reference plasmids (G1a H77 and G1b Con1), the error rates (either by HCV region or by HCV subtype) were found to be consistent with those described in the literature. [46]–[48] Subsequently, by combining the specific error rates of our UDPS methodology and a Poisson distribution, we were able to establish a conservative cut-off (≥0.5%) which minimized false positive results in our HCV variants analysis.

In conclusion, the study reported here provide a rich source of data on the abundance and prevalence of HCV variants in treatment-naive G1 patients, and provide insights into the possible factors contributing to the observed differences between G1a- and G1b-infected patients in virological response and breakthrough rates. Using the largest collection of UDPS data of HCV isolates from treatment-naïve patients to date, we found that, at the genetic level, the variability was similar in G1a and G1b isolates and in both NS3 and NS5B regions. We observed no clear difference in entropy between G1a and 1b HCV, at least in the HCV regions studied here; the number of naturally occurring high-abundance and low-abundance drug-resistant variants in NS5B was similar in G1a and G1b. However, a non-significant but higher prevalence of PI-resistant low-abundance variants and a significantly higher prevalence of high-abundance PI-resistant variants were observed in G1a than G1b NS3 samples. Importantly, this large-scale study strongly supports the elimination of higher genetic variability in G1a as a major contributing factor to the observed differences in virological response and breakthrough rates between G1a- and G1b-infected patients treated with PIs. Instead, a natural prediction emerging from our results is that factors unrelated to intrinsic genetic variability, such as random mutagenesis and a low genetic barrier to resistance, are more likely to play major roles in determining a patient’s response to DAA-based therapy. These factors may be considered preferentially in the development and clinical testing of future DAA-based therapy, which has the potential of becoming interferon-free and all-oral regimens that will cure most patients regardless of HCV genotypes, subtypes and prior treatment status [49].

## Supporting Information

File S1 Lists of primers and barcodes used to generate the 454 amplicons.
(DOCX)
