# Supplementary material for: Similar Prevalence of Low-Abundance Drug-Resistant Variants in Treatment-Naive Patients with Genotype 1a and 1b Hepatitis C Virus Infections as Determined by Ultradeep Pyrosequencing
Source: PLoS One. 2014 Aug 20;9(8):e105569. doi: 10.1371/journal.pone.0105569 (PMC4139363; doi:10.1371/journal.pone.0105569)
Supplement: File S1 — Lists of primers and barcodes used to generate the 454 amplicons. (DOCX) [file pone.0105569.s001.docx]

**Table S1.** Listing of primers used to generate the 454 amplicons

| **Primer Name** | **Sequence 5’- 3’** |  | **NT position** | **AA position** | **Amplicon Name** | | | |
| --- | --- | --- | --- | --- | --- | --- | --- | --- |
| **First round of PCR – Genotype 1a – NS3** | | | | | | |  |  |
| NS3F71A | GGGACAARAACCARGTGGAG |  | 71-90 | 23-30 | NS3–PCR1 | | | |
| NS3R821A | AGRAAYTTGCCGTAGGTGGA |  | 821-802 | 273-267 |  |  |  |  |
| **First round of PCR – Genotype 1a – NS5B** | | | | | | |  |  |
| NS5BF709A | GAGGCAATYTACCARTGTTGTGA |  | 709-731 | 236-243 | NS5B–PCR1 | | | |
| NS5BR1672A | GACACGCTGTGATAAATGTCTCC |  | 1694-1672 | 564-557 |  |  |  |  |
| **Second round of PCR – Genotype 1a – NS3** | | | | | | |  |  |
| NS3F71A | 454adapterA-MID-GGGACAARAACCARGTGGAG |  | 71-90 | 23-30 | NS3-A1 | | | |
| NS3R327A | 454adapterB-MID-CCTCGTGACCARGTARAGGT |  | 327-308 | 109-102 |  |  |  |  |
| NS3F203A | 454adapterA-MID-ARGGTCCTGTYATCCAGATGTA |  | 203-224 | 67-74 | NS3-A2 | | | |
| NS3R548A | 454adapterB-MID-ACCGGGGAYCTCATRGTTGT |  | 548-528 | 182-176 |  |  |  |  |
| **Second round of PCR – Genotype 1a – NS5B** | | | | | | |  |  |
| NS5BF709A | 454adapterA-MID-GAGGCAATYTACCARTGTTGTGA |  | 709-731 | 236-243 | NS5B-A1 | | | |
| NS5BR1033A | 454adapterB-MID-TGGTCATRGCCTCCGTGAAG |  | 1033-1014 | 344-338 |  |  |  |  |
| NS5BF902A | 454adapterA-MID-CAGCCTGTCGAGCCGCAG |  | 902-919 | 300-306 | NS5B-A2 | | | |
| NS5BR1210A | 454adapterB-MID-GAGTGTGTYTTGCTGTYTCCCAC |  | 1210-1188 | 403-396 |  |  |  |  |
| NS5BF1139A | 454adapterA-MID-GGGTCTAYTACCTYACCCGTGAC |  | 1139-1161 | 379-387 | NS5B-A3 | | | |
| NS5BR1507A | 454adapterB-MID-GGTGTCTCCARGYTCGCAAG |  | 1507-1488 | 502-496 |  |  |  |  |
| **First round of PCR – Genotype 1b – NS3** | | | | | | |  |  |
| NS3F70B | CGGGACARGAACCARGTCG |  | 70-88 | 23-29 | NS3–PCR1 | | | |
| NS3R876B | GCACTCATCRCAYATTATGATGTC |  | 876-853 | 292-284 |  |  |  |  |
| **First round of PCR – Genotype 1b – NS5B** | | | | | | |  |  |
| NS5BF706B | GAGGAGTCAATHTACCAATGTTGTG |  | 706-730 | 235-243 | NS5B–PCR1 | | | |
| NS5BR1622B | GCYGGRATTGGAGTGAGTTTRAG |  | 1622-1600 | 540-533 |  |  |  |  |
| **Second round of PCR – Genotype 1b – NS3** | | | | | | |  |  |
| NS3F70B | 454adapterA-MID-CGGGACARGAACCARGTCG |  | 70-88 | 23-29 | | NS3-A1 | |  |
| NS3R314B | 454adapterB-MID-TAAAGGTCYGARCTGCCGCA |  | 314-295 | 104-98 | |  |  |  |
| NS3F237B | 454adapterA-MID-CCARGACCTCGTCGGCTG |  | 237-254 | 79-84 | | NS3-A2 | |  |
| NS3R593B | 454adapterB-MID-TGRAATGTYTGCGGTACGG |  | 593-575 | 197-191 | |  |  |  |
| **Second round of PCR – Genotype 1b – NS5B** | | | | | | |  |  |
| NS5BF706B | 454adapterA-MID-GAGGAGTCAATHTACCAATGTTGTG |  | 706-730 | 235-243 | NS5B-A1 | | | |
| NS5BR1037B | 454adapterB-MID-TACCTRGTCATRGCCTCCGTG |  | 1037-1017 | 345-339 |  |  |  |  |
| NS5BF914B | 454adapterA-MID-CTGCRAAGCTCCRGGACTG |  | 914-932 | 304-310 | NS5B-A2 | | | |
| NS5BR1229B | 454adapterB-MID-CCTAGCCAGGARTTRACTGGA |  | 1229-1209 | 409-403 |  |  |  |  |
| NS5BF1184B | 454adapterA-MID-CTGCGTGGGARACAGCTAGAC |  | 1184-1204 | 394-401 | NS5B-A3 | | | |
| NS5BR1508B | 454adapterB-MID-CGATGTCTCCAGRCYCGCAA |  | 1507-1489 | 502-496 |  |  |  |  |

**Table S2.** List of barcodes used to generate the 454 amplicons

| **Barcode ID** | **Nucleotide Sequence** | **Amplicon** | **HCV Genotype** | **Barcode ID** | **Nucleotide Sequence** | **Amplicon** | **HCV Genotype** |
| --- | --- | --- | --- | --- | --- | --- | --- |
| **BC1** | CGTAGCA | NS5B-A2 + NS5B-A3 | 1a + 1b | **BC14** | ACGACAT | NS3-A1 + NS3-A2 | 1a |
| **BC2** | ACGCTGT | NS5B-A2 + NS5B-A3 | 1a + 1b | **BC15** | CACGTAT | NS3-A1 + NS3-A2 | 1a |
| **BC3** | AGCTCGT | NS5B-A2 + NS5B-A3 | 1a + 1b | **BC16** | TACTCAT | NS3-A1 + NS3-A2 | 1a |
| **BC4** | ATCGTCT | NS5B-A2 + NS5B-A3 | 1a + 1b | **BC17** | AGCAGCT | NS3-A1 + NS3-A2 | 1a |
| **BC5** | CAGTCGT | NS5B-A2 + NS5B-A3 | 1a + 1b | **BC18** | CAGAGAT | NS3-A1 + NS3-A2 | 1a |
| **BC6** | CATCGCT | NS5B-A2 + NS5B-A3 | 1a + 1b | **BC19** | TCATCGT | NS3-A1 + NS3-A2 | 1a |
| **BC7** | CGACACA | NS5B-A2 + NS5B-A3 | 1a + 1b | **BC20** | ACTGCGT | NS3-A1 + NS3-A2 | 1a |
| **BC8** | CGTGACT | NS5B-A2 + NS5B-A3 | 1a + 1b | **BC21** | CGTACAT | NS3-A1 + NS3-A2 | 1a |
| **BC9** | CTGCTGA | NS5B-A2 + NS5B-A3 | 1a + 1b | **BC22** | TATATAT | NS3-A1 + NS3-A2 | 1a |
| **BC10** | TAGAGTA | NS5B-A2 + NS5B-A3 | 1a + 1b | **BC23** | AGACGAT | NS3-A1 + NS3-A2 | 1a |
| **BC11** | TAGCAGT | NS5B-A2 + NS5B-A3 | 1a + 1b | **BC24** | CTAGAGT | NS3-A1 + NS3-A2 | 1a |
| **BC12** | TATCATA | NS5B-A2 + NS5B-A3 | 1a + 1b | **BC25** | TGAGCAT | NS3-A1 + NS3-A2 | 1a |
| **BC13** | TGACTGA | NS5B-A2 + NS5B-A3 | 1a + 1b | **BC26** | AGAGTGT | NS3-A1 + NS3-A2 | 1a |
| **BC40** | ACAGCTC | NS5B-A1 | 1a + 1b | **BC27** | ACACAGA | NS3-A1 + NS3-A2 | 1b |
| **BC41** | ACGTATC | NS5B-A1 | 1a + 1b | **BC28** | CACAGTG | NS3-A1 + NS3-A2 | 1b |
| **BC42** | ACTATCA | NS5B-A1 | 1a + 1b | **BC29** | TACGACG | NS3-A1 + NS3-A2 | 1b |
| **BC43** | AGCACTC | NS5B-A1 | 1a + 1b | **BC30** | AGATGCA | NS3-A1 + NS3-A2 | 1b |
| **BC44** | AGCGTAC | NS5B-A1 | 1a + 1b | **BC31** | CATGATG | NS3-A1 + NS3-A2 | 1b |
| **BC45** | CAGCTAC | NS5B-A1 | 1a + 1b | **BC32** | TCTGCTG | NS3-A1 + NS3-A2 | 1b |
| **BC46** | CATACTC | NS5B-A1 | 1a + 1b | **BC33** | AGCGACA | NS3-A1 + NS3-A2 | 1b |
| **BC47** | CGAGCTA | NS5B-A1 | 1a + 1b | **BC34** | CACTCTA | NS3-A1 + NS3-A2 | 1b |
| **BC48** | CGTCGTA | NS5B-A1 | 1a + 1b | **BC35** | TAGATCG | NS3-A1 + NS3-A2 | 1b |
| **BC49** | TACTGTC | NS5B-A1 | 1a + 1b | **BC36** | ACTGATA | NS3-A1 + NS3-A2 | 1b |
| **BC50** | TAGTACA | NS5B-A1 | 1a + 1b | **BC37** | CAGCACG | NS3-A1 + NS3-A2 | 1b |
| **BC51** | TGATATC | NS5B-A1 | 1a + 1b | **BC38** | TGCTAGA | NS3-A1 + NS3-A2 | 1b |
| **BC52** | TGTACTA | NS5B-A1 | 1a + 1b | **BC39** | AGAGATG | NS3-A1 + NS3-A2 | 1b |
